# Public understanding of the purpose of cancer screening: A population-based survey

**DOI:** 10.1177/0969141317699440

**Published:** 2017-05-22

**Authors:** Amanda J Chorley, Yasemin Hirst, Charlotte Vrinten, Christian von Wagner, Jane Wardle, Jo Waller

**Affiliations:** Research Department of Behavioural Science and Health, UCL, London, UK

**Keywords:** Cancer screening, informed choice, public awareness, cancer knowledge, cancer prevention, early diagnosis

## Abstract

**Objectives:**

In examining informed choice in cancer screening, we investigated public awareness that some screening programmes aim to prevent cancer, while others seek to detect cancer at an early stage.

**Methods:**

A population-based survey of adults aged 50–70 in England (n = 1433), including data on demographic characteristics and screening experience. Participants were asked to select the main purpose of cervical, breast, and colorectal cancer screening (both faecal occult blood testing and flexible sigmoidoscopy).

**Results:**

Across all four screening programmes, most people thought the main aim was to catch cancer early (71–78%). Only 18 and 14% knew that cervical screening and flexible sigmoidoscopy, respectively, are primarily preventive. Knowledge of the preventive aspect of these two programmes was low across the board, with few demographic patterns. By contrast, 78 and 73% of the sample were aware that breast screening and the faecal occult blood test, respectively, predominantly aim to detect cancer early. For these programmes, accurate knowledge was socially graded, lower in ethnic minority groups, and positively associated with previous participation in the programmes.

**Conclusions:**

Our findings suggest that although awareness of the purpose of early detection screening is high, awareness that screening can prevent cancer is low across all demographic groups. Understanding the purpose of screening is a key aspect of informed choice but despite current communication strategies highlighting these differences, people do not seem to have a nuanced understanding of these differing aims. Our findings may be indicative of a broader public scepticism about the preventability of cancer.

## Introduction

As part of a broader movement to involve patients in their health care, in recent years there has been a shift in focus within communication about cancer screening. Previous attempts to maximise attendance through emphasising the benefits of screening have been replaced by efforts to enable individuals to make an informed choice, through the provision of comprehensive information.^1^ Although screening is a major public health element of cancer control, as with any public health initiative, while some individuals will gain tremendous benefit through participation, the majority of people will see no benefit at all. Some people will also be exposed to harms, including the physical and psychological effects of overdiagnosis and overtreatment, of false positive results and subsequent investigations, and potential delay in seeking help if a person experiences cancer symptoms after a negative result.^2^

In line with the increasing emphasis on individuals making an informed choice about cancer screening, there has been a growing concern regarding the extent to which people are aware of and understand these limitations and risks. Research has shown that general enthusiasm for screening is high, with the majority of people endorsing the idea that cancer screening for healthy individuals is ‘almost always a good idea’.^3,4^ Other research, mainly in the breast screening context, has shown that many people are unaware of overdiagnosis,^4–7^ or find the concept confusing,^8^ but once they understand it, feel that it is an important factor to take into account in screening decisions. However, even when the concept is described, many people would still want to know if they had a cancer that may not cause them any harm within their lifetime^3,4^ or state that the information did not change their screening intentions.^6–8^ Even in situations where individuals have experienced a false positive result, they remain overwhelmingly positive about screening.^3,5^

Because of these findings, the majority of work on supporting informed choice about screening has focused on ways to accurately and accessibly convey information on limitations and risks. However, for individuals to make a truly informed decision regarding screening, it is important that they not only understand the potential harms and benefits, but also the purpose of screening,^9^ including whether the screening programme in question is predominantly designed for early detection or to prevent cancer. Although there is not always a clear-cut distinction between screening tests aimed at prevention and those aimed at early detection, the information provided with screening invitations in England clearly identifies differing aims for different programmes. Of the three cancer screening programmes in operation, cervical and bowel scope screening (a once-only flexible sigmoidoscopy (FS) offered as part of the bowel cancer screening programme) are described as preventing cancer through the detection, and subsequent removal, of pre-cancerous lesions and polyps, respectively. Breast screening and guaiac faecal occult blood tests (gFOBt; also offered as part of the bowel cancer screening programme) are described as aiming to detect disease early, when treatment is more effective. Despite the NHS information leaflets accompanying invitations making the aim of each screening programme explicit,^10–13^ it is not clear to what extent people understand these differences.

This study sought to establish whether middle aged and older adults in the general population are aware that some cancer screening programmes aim to prevent cancer and others aim to detect cancer early. A secondary objective was to explore demographic correlates of accurate knowledge of the purpose of four types of cancer screening and whether accurate knowledge was associated with screening experience.

## Methods

Data were collected in England between March and April 2015 as part of the Attitudes and Behaviour about Cancer UK Survey, a population-based survey of adults aged 50–70, delivered via the TNS Global Ltd omnibus. Sample points, defined using 2011 Census small area statistics and the Postcode Address File, were selected using stratified random location sampling. Within each location, quotas were set for age, gender, working status, and presence of children in the home. The survey was conducted using home-based computer-assisted personal interviewing. The measures reported here were part of a larger module that included questions on a range of factors related to cancer screening. This survey was exempt from UCL ethical committee approval requirements.

Respondents were given the following statement: ‘Some cancer screening tests are designed mainly to find cancer early. Others are designed to find and treat pre-cancer (abnormal cells that aren’t yet cancer) and prevent cancer from developing’. They were then asked ‘For each of these tests, could you say whether you think it’s mainly to find cancer early or to prevent it?’, and given the response options: mainly to find cancer; mainly to prevent cancer; heard of this test but don’t know what it is (specifically) designed for; never heard of this test; don’t know. A binary variable was created for each screening programme with the categories ‘accurate knowledge’ and ‘inaccurate knowledge’, with ‘mainly to prevent cancer’ coded as an accurate response for cervical and FS screening, and ‘mainly to detect cancer early’ coded as an accurate response for breast screening and gFOBt.

Respondents were asked whether they had ever been invited to participate in each screening programme, and if so, whether they had attended for screening (or had completed the stool test kit in the case of gFOBt). These questions were only asked to respondents who met the inclusion criteria for the given programme, in terms of their age and sex (Box 1). Women in our sample aged 65–70 no longer meet the eligibility criteria for the cervical screening programme and were therefore not asked the invitation question. They were therefore excluded from the ‘invitation’ analyses of cervical screening, as were men aged 65–70, to ensure comparability within the subsample on age.

**Box 1.** Eligibility criteria for each screening programme within our sample.

• *Cervical screening:* Women aged 50–64	
• *Breast screening:* Women aged 50–70	
• *gFOBt:* Men and women aged 60–70	
• *FS:* Men and women aged 55–60	

Participants who gave ‘Don’t know’ responses to the cancer screening experience items were excluded from analysis. Participants were also excluded from programme-specific analyses if they reported a previous diagnosis of the type of cancer in question.

Age, gender, marital status (married or cohabiting; single; separated, widowed, or divorced), ethnicity (white; non-white), education (degree or higher; A-Levels, GCSEs, ONC or BTEC; no qualifications), and social grade were recorded for each respondent. Social grade was classified according to the National Readership Survey system^14^ and is based on occupation: A/B (managerial/ professional), C1 (supervisory), C2 (skilled manual), DE (semi-skilled or unskilled manual/pensioners/casual workers/unemployed). Participants were also asked whether they had ever been diagnosed with breast, cervical, or colorectal cancer.

Data were analysed in SPSS 21.0.^15^ Sample characteristics, including knowledge of the purpose of each screening programme, were described. Demographic predictors of accurate knowledge within the total population were examined for each screening programme using univariate regression models, as were the associations between screening experience (invitation and participation) and accurate knowledge.

Up to a total of three multivariable logistic regression models were carried out for each screening programme. Model 1 included all demographic variables and was carried out for each of the four screening programmes. The additional models controlled for demographic variables and included invitation status (Model 2) or attendance status (Model 3). Model 3 did not control for invitation status, as only participants who had been invited were asked about participation.

## Results

In the sample of 1433 individuals aged 50–70 (mean age 60.3; *SD* = 6.29), just over half of respondents were female (50.5%), and the majority were from white ethnic backgrounds (93.1%; c.f. 92.4% for this age group in the 2011 census^16^). Most respondents were married (61.8%), while 23.6% were separated, widowed, or divorced, and 14.6% were single. Social grades D/E contained the largest percentage of respondents (36.1%), while grades A/B, C1, and C2 represented 20.2, 23.0, and 20.8% of respondents, respectively. Almost a third of respondents had post-school qualifications (31.5%), 40.1% had GCSEs or A-levels, and 28.4% had no educational qualifications (Table 1).

**Table 1.** Sample characteristics.

	n	%
Age		
50–54	351	24.5
55–59	276	19.3
60–64	354	24.7
65–70	452	31.5
Gender		
Male	710	49.5
Female	723	50.5
Social Grade		
A/B	289	20.2
C1	329	23.0
C2	298	20.8
D/E	517	36.1
Education		
No qualifications	374	28.4
GCSE/A-Levels	527	40.1
Degree or higher	414	31.5
Ethnicity		
White	1330	93.1
Non-white	99	6.9
Marital status		
Married	886	61.8
Single	209	14.6
Previously married	338	23.6
Cervical screening		
Eligible	496	35.0
Ever invited (of eligible)	477	97.9
Ever participated (of invited)	425	89.1
Flexible sigmoidoscopy		
Eligible	273	19.2
Ever invited (of eligible)	32	11.8
Ever participated (of invited)	22	68.8
Guaiac faecal occult blood test		
Eligible	798	56.2
Ever invited (of eligible)	678	85.3
Ever participated (of invited)	535	79.3
Breast screening		
Eligible	694	49.4
Ever invited (of eligible)	680	98.6
Ever participated (of invited)	613	90.1

From our sample, of the population eligible for cervical screening (women aged 50–64; n = 496), 97.9% (n = 477) reported having received at least one cervical screening invitation. Of these, 89.1% had participated at least once. Among the eligible FS population (n = 273), 11.8% (n = 32) had received an invitation, of whom 68.8% had participated. From those men and women aged 60–70 (n = 798) eligible for gFOBt screening, 85.3% (n = 678) reported having received at least one stool test kit, and of these 79.3% had completed it at least once. Among women aged 50–70 (n = 694) eligible for breast screening, 98.6% (n = 680) reported having received at least one invitation, and of these 90.1% had participated at least once.

As shown in Table 2, over 70% of participants believed that each of the four screening tests had a primary purpose of detecting cancer early. The two programmes aimed at early detection (breast and gFOBt) had the highest proportion of the sample accurately identifying their purpose (77.9 and 73.2%, respectively). Only 17.6% were able to identify prevention as the primary purpose of cervical screening, and for FS only 13.8%. Among respondents who answered all four knowledge questions (n = 1426), only 13 individuals (0.9%) had accurate knowledge of the purpose of all the screening programmes, while 99 participants (6.9%) were able to identify the purpose of three of the screening programmes. The majority (73.0%) had accurate knowledge of the purpose of two of the screening programmes, while 12.2% (n = 174) of the sample were able to correctly identify the purpose of one of the screening programmes. A further 99 individuals (6.9%) did not correctly identify the purpose of any of the programmes.

**Table 2.** Knowledge of the purpose of each screening programme.

	Cervical (n = 1043)		FS (n = 1431)		gFOBT (n = 1430)		Breast (n = 1428)	
	n	%	n	%	N	%	n	%
Early detection	1043	72.9	1019	71.2	1047	**73.2**	1113	**77.9**
Prevention	251	**17.6**	197	**13.8**	211	14.8	194	13.6
Don’t know	136	9.5	215	15.0	172	12.0	121	8.5

Percentage of respondents with accurate knowledge are presented in bold. FS: flexible sigmoidoscopy; gFOBt: guaiac faecal occult blood test.

For cervical screening we found no significant association between invitation status, participation, or any of the demographic characteristics and accurate knowledge of the purpose of screening, in either univariate or multivariate logistic regression analyses (Table 3).

**Table 3.** Predictors of accurate knowledge of the aim of each screening programme.

	Cervical screening		Flexible sigmoidoscopy		gFOBt		Breast screening	
	OR (95% CI)	P value	OR (95% CI)	P value	OR (95% CI)	P value	OR (95% CI)	P value
Age^a^		0.58		0.27		0.002		0.06
50–54	1.00 (ref)	(ref)	1.00 (ref)	(ref)	1.00 (ref)	(ref)	1.00 (ref)	(ref)
55–59	1.18 (0.77–1.81)	0.44	1.23 (0.78–1.96)	0.38	0.79 (0.55–1.14)	0.20	0.76 (0.51–1.13)	0.17
60–64	0.95 (0.63–1.43)	0.80	0.88 (0.55–1.40)	0.59	1.27 (0.88–1.83)	0.20	0.80 (0.55–1.19)	0.27
65–70	–	–	0.78 (0.50–1.24)	0.30	**1.56 (1.09–2.24)**	**0.01**	1.21 (0.82–1.78)	0.34
Gender^a^		0.11		0.80		0.19		0.01
Male	1.00 (ref)	(ref)	1.00 (ref)	(ref)	1.00 (ref)	(ref)	1.00 (ref)	(ref)
Female	1.32 (0.94–1.87)	0.11	1.04 (0.75–1.45)	0.80	1.19 (0.92–1.53)	0.19	**1.48 (1.13–1.95)**	**0.01**
Social grade^a^		0.43		0.99		0.11		0.08
D/E (low)	1.00 (ref)	(ref)	1.00 (ref)	(ref)	1.00 (ref)	(ref)	1.00 (ref)	(ref)
C2	1.31 (0.79–2.19)	0.30	0.96 (0.61–1.51)	0.85	1.18 (0.83–1.67)	0.37	1.20 (0.83–1.74)	0.33
C1	1.24 (0.75–2.05)	0.40	0.94 (0.59–1.49)	0.79	1.40 (0.98–2.02)	0.07	**1.71 (1.14–2.57)**	**0.01**
A/B (high)	1.59 (0.91–2.76)	0.10	0.90 (0.53–1.53)	0.71	**1.65 (1.08–2.52)**	**0.02**	1.28 (0.82–2.00)	0.28
Education^a^		0.81		0.57		0.22		0.21
No qualifications	1.00 (ref)	(ref)	1.00 (ref)	(ref)	1.00 (ref)	(ref)	1.00 (ref)	(ref)
GCSEs/A-Levels	1.08 (0.67–1.74)	0.75	1.23 (0.81–1.87)	0.32	0.89 (0.65–1.22)	0.45	1.24 (0.89–1.73)	0.20
Higher education	1.19 (0.69–2.05)	0.53	1.07 (0.65–1.77)	0.79	1.19 (0.80–1.77)	0.38	1.44 (0.95–2.18)	0.09
Ethnicity^a^		0.87		0.27		0.004		0.03
White	1.00 (ref)	(ref)	1.00 (ref)	(ref)	1.00 (ref)	(ref)	1.00 (ref)	(ref)
Non-white	1.06 (0.55–2.01)	0.87	1.39 (0.77–2.52)	0.27	**0.51 (0.32–0.80)**	**0.004**	**0.59 (0.36–0.96)**	**0.03**
Marital status^a^		0.12		0.05		0.92		0.42
Married	1.00 (ref)	(ref)	1.00 (ref)	(ref)	1.00 (ref)	(ref)	1.00 (ref)	(ref)
Single	0.98 (0.58–1.67)	0.95	1.12 (0.69–1.84)	0.64	0.99 (0.68–1.44)	0.94	0.80 (0.54–1.18)	0.25
Previously married	1.51 (1.00–2.29)	0.05	**1.62 (1.11–2.38)**	**0.01**	0.94 (0.68–1.28)	0.68	0.85 (0.61–1.19)	0.35
Invited^b^		0.68		0.79		<0.001		0.14
No	1.00 (ref)	(ref)	1.00 (ref)	(ref)	1.00 (ref)	(ref)	1.00 (ref)	(ref)
Yes	1.57 (0.19–13.13)	0.68	1.15 (0.41–3.26)	0.79	**2.54 (1.62–3.99)**	** <0.001**	3.22 (0.70–14.94)	0.14
Participated^c^		0.95		0.75		0.03		<0.001
No	1.00 (ref)	(ref)	1.00 (ref)	(ref)	1.00 (ref)	(ref)	1.00 (ref)	(ref)
Yes	0.98 (0.45–2.12)	0.95	0.55 (0.01–22.90)	0.75	**1.71 (1.07–2.74)**	**0.03**	**3.49 (1.85–6.58)**	**<0.001**

gFOBt: guaiac faecal occult blood test. Values indicated in bold are significant at p < 0.05.

a Mutually adjusted for all other socio-demographic characteristics.

b Adjusted for all socio-demographic characteristics, but not for participation.

c Adjusted for all socio-demographic characteristics, but not for invitation status.

We found that for FS in univariate analyses, only marital status was significantly associated with accurate knowledge, with separated, widowed, or divorced participants more likely to have accurate knowledge than the married reference group (*OR = *1.57, 95% CI 1.12–2.22). This remained significant after adjusting for all other demographic predictors (*OR = *1.62, 95% CI 1.11–2.38). Models including screening experience revealed no association between accurate knowledge and invitation status or participation (Table 3).

For gFOBt, in unadjusted analyses, being older (aged 65–70; *OR = *1.56, 95% CI 1.13–2.15), being affluent (Social Grade AB; *OR = *1.97, 95% CI 1.39–2.80, or Social Grade C1; *OR* = 1.38, 95% CI 1.01–1.88), having previously received a stool kit (*OR = *1.94, 95% CI 1.52–2.47), and having completed a stool kit (*OR* = 1.66, 95% CI 1.08–2.56) were significantly associated with accurate knowledge. Individuals from a non-white ethnic background had lower odds of accurate knowledge (*OR* = 0.45, 95% CI 0.29–0.68). The demographic predictors remained significant in Model 1, which mutually adjusted for all demographic variables (Table 3), with the exception of Social Grade C1 (*OR* = 1.40, 95% CI 0.98–2.02). In Model 2, which included all demographic variables as well as invitation status, being invited had the strongest effect on having accurate knowledge of the purpose of gFOBt (*OR = *2.54, 95% CI 1.62–3.99). In this model, only being in social grades AB (*OR = *1.69, 95% CI 1.10–2.59) was associated with higher odds of accurate knowledge. Being aged 60–64 (*OR = *0.60, 95% CI 0.36–0.99) and from a non-white ethnic background (*OR = *0.55, 95% CI 0.35–0.88) were negatively associated with accurate knowledge. With the inclusion of participation status in Model 3, no demographic characteristics remained predictive of accurate knowledge of the purpose of gFOBt. However, having completed a kit was predictive of accurate knowledge (*OR* = 1.71, 95% CI 1.07–2.74) in this model.

In relation to breast screening, in unadjusted analyses, being female (*OR = *1.34, 95% CI 1.04–1.73), being more affluent (Social Grades AB (*OR = *1.68, 95% CI 1.18–2.40) or C1 (*OR = *1.94, 95% CI 1.36–2.77)), and having a degree (*OR = *1.60, 95% CI 1.14–2.25) were associated with accurate knowledge of the purpose of screening, whereas individuals from non-white ethnic backgrounds had lower odds of having accurate knowledge compared with those from white backgrounds (*OR = *0.56, 95% CI 0.36–0.86). Having been invited (*OR = *1.37, 95% CI 1.06–1.77) and having participated (*OR = *3.10, 95% CI 1.82–5.31) in breast screening were significantly associated with accurate knowledge of its purpose. Neither age nor marital status was associated with knowledge.

Once demographic factors had been mutually adjusted in Model 1, being female (*OR* = 1.48, 95% CI 1.13–1.95) and from social grade C1 (*OR* = 1.71, 95% CI 1.14–2.57) remained significantly associated with having accurate knowledge (Table 3). Individuals from a non-white ethnic background remained significantly less likely to have accurate knowledge (*OR* = 0.59, 95% CI 0.36–0.96). After adjusting for demographic characteristics, invitation status was no longer significantly associated with accurate knowledge. Being from social grade C1 (*OR* = 1.74, 95% CI 1.16–2.61) and from a non-white ethnic background (*OR = *0.60, 95% CI 0.37–0.98) remained significantly associated with accurate knowledge, in the same direction as shown in Model 1. In Model 3, individuals aged 55–59 (*OR* = 0.51, 95% CI 0.26–0.99) or 60–64 (*OR* = 0.48, 95% CI 0.25–0.90), and those from a non-white ethnic background (*OR = *0.36, 95% CI 0.17–0.77) were significantly less likely to have accurate knowledge. The differences between socioeconomic grades were no longer significant. The strongest predictor of accurate knowledge was participation in screening, with those who had participated at least once being 3.49 times more likely to have accurate knowledge (95% CI 1.85–6.58).

## Discussion

Our findings strongly suggest that the general population think of screening as a means of detecting cancer early, with only a minority being aware of the potential of certain cancer screening programmes to prevent cancer by identifying and treating its precursor lesions. Beliefs were similar across screening programmes, with little evidence that people distinguish between the aims of the different programmes, despite them being clearly stated in NHS information leaflets. For the Bowel Scope (FS) programme, which is still in the roll-out phase, this is perhaps unsurprising, but low awareness that cervical screening has a preventive aspect is more striking, given that the programme has been running since 1988, with 75–80% of women participating on a regular basis. Within our sample, previous invitation to, or participation in cervical screening was not associated with accurate knowledge about its purpose. Our findings suggest that despite the development of NHS information materials designed to facilitate informed choice, the purpose of screening is not being successfully communicated. This may be due to the far more dominant public discourse surrounding the importance of early detection of cancer and the role of screening programmes in this.

Poor awareness of the preventive purpose of cervical screening is a finding consistent with previous studies, which often find women receiving pre-cancerous results believe they have cancer.^17,18^ This belief contributes to the adverse psychological consequences of an abnormal result, which further emphasises the need to ensure that the purpose of screening is understood before people take part.

It is possible that misunderstanding about the purpose of screening may also affect people’s decisions about participation. It could be hypothesised that people with high levels of cancer fear may avoid taking part in screening,^19–21^ for fear of a diagnosis, and that this could be alleviated if the preventive aim of some of the programmes were better understood. This may be especially important for FS screening, as uptake of this primarily preventive screening test is well below that of the other screening programmes (43.1%).^22^

The extent to which a better understanding of the preventive aim of certain screening programmes would alleviate these issues is unclear. However, in the case of ductal carcinoma in situ there is evidence that the use of terms not containing the words *cancer* or *carcinoma* has an impact on women’s concern and treatment preferences.^23,24^ This suggests that a good understanding that cervical screening and FS aim to remove abnormalities that are pre-cancerous may have an impact on both the psychological consequences of abnormal results and on behavioural intentions. However, given that the NHS information leaflets already clearly state these aims, it is unclear what changes could be made to increase engagement with and understanding of the information.

Our study is not without limitations. Although we measured ‘ever’ uptake of each screening programme, and would therefore expect levels of participation to be higher than routine uptake figures, it is likely that our sample was somewhat more engaged with cancer screening than the general population. If this were the case, it may suggest that we overestimated public understanding of the purpose of screening, at least for breast screening and gFOBT, which both showed significant associations between participation and accurate knowledge. It is also possible that, due to the nature of the Omnibus survey design, participants will have been prompted to consider cancer screening to a greater extent than they usually would during daily life. We do not know the extent to which the previous survey questions may have influenced individual responses, and this was not assessed with other knowledge-based questions. Likewise, population-based sampling meant that the sample size for FS is smaller compared with other groups, which subsequently reduced the robustness of the statistical models for FS. Both limitations may be addressed in future research, with a greater sample size and through the development of a validated informed choice survey. Finally, our analysis is based on the primary purpose of screening as it is stated in the NHS information materials and is therefore somewhat overly simplistic. It does not take account of the fact that both cervical and FS screening can detect cancer as well as preventing it through the detection of precancerous lesions or that breast and gFOBt screening could be seen as aiming to prevent late stage disease.

## Conclusions

The early detection benefits of cancer screening have long been at the forefront of campaigns to encourage screening participation. With the recent emphasis on informed choice, NHS leaflets for breast, cervix, and colorectal screening now include information on screening harms and on the primary purpose of each programme. Our findings suggest that despite this, the distinction between prevention and early detection is not clear in the minds of the public, in particular, the potential for some of these tests to prevent cancer. More positively, the message that screening can detect cancer at an earlier stage appears to be very effectively communicated, leading to a high level of awareness of this aspect of screening.
